# A review of population-based prevalence studies of physical activity in adults in the Asia-Pacific region

**DOI:** 10.1186/1471-2458-12-41

**Published:** 2012-01-17

**Authors:** Rona Macniven, Adrian Bauman, Marian Abouzeid

**Affiliations:** 1 Prevention Research Collaboration, School of Public Health, University of Sydney, Sydney, Australia; 2 Greater Green Triangle University Department of Rural Health, Flinders and Deakin Universities, Warrnambool, Australia

## Abstract

**Background:**

Physical activity (PA) surveillance is an important component of non-communicable disease risk factor monitoring, and occurs through national and international surveillance systems. This review identifies population PA estimates for adults in the Asia-Pacific region, and examines variation in trends and prevalence rates obtained using different PA measures.

**Methods:**

Data were obtained from a MEDLINE search; World Health Organization's Global Health Infobase; Government websites and reference lists of relevant papers. Inclusion criteria included: national studies or those reporting large scale population-level data; data published from 2000 to 2010 and trend data prior; sample sizes over n = 1000, or fewer subjects in small nations.

**Results:**

In total, 56 population surveys from 29 Asia-Pacific countries were identified. Data on 'sufficient physical activity' amongst adults were available from 45 studies (80%), with estimates ranging from 7% to 93% (median 62%, inter-quartile range 40%-85%). For 14 countries, estimates of 'sufficient activity' were documented in multiple surveys using different methods, with the largest variation from 18% to 92% in Nepal. Median or mean MET-minutes/day, reported in 20 studies, ranged from 6 to 1356. Serial trend data were available for 11 countries (22%), for periods spanning 2-10 years. Of these, five countries demonstrated increases in physical activity over time, four demonstrated decreases and three showed no changes.

**Conclusions:**

Many countries in the Asia-Pacific region collect population-level PA data. This review highlights differences in estimates within and between countries. Some differences may be real, others due to variation in the PA questions asked and survey methods used. Use of standardized protocols and measures, and combined reporting of data are essential goals of improved international PA surveillance.

## Background

Regular physical activity is an important aspect of non-communicable disease (NCD) prevention [1,2] and inactivity is the fourth largest contributor to global mortality and morbidity [3-5]. Physical activity is undertaken in several domains of daily life, including work, transport and leisure time [6]. This distinction is especially important in developing and transitional countries, where recreational physical activity makes a smaller contribution to total energy expenditure than occupational or transportation related activity [7,8]. The measurement of population levels of physical activity is necessary to guide health promotion initiatives and policy formulation and to assess the impact of large-scale policies and programs designed to increase activity [9]. For adults, physical activity is usually assessed by self-report, through interviews or self-administered questionnaires. The concept of 'sufficient' activity relates to the proportion of the population achieving recommended levels of physical activity for promoting health and preventing disease [10].

In countries such as the United States, Finland, and Canada, standardized population-level physical activity surveillance in adults has occurred for several decades [11-13]. In other countries, surveillance systems have been less consistent and have asked different physical activity questions and are therefore not comparable [12]. Physical activity instruments vary with regard to the questions asked; recall periods and the areas of daily life assessed [9]. Many surveys in developed countries explore leisure time activities only. These may yield very different prevalence estimates compared to newer international questionnaires that examine a wider range of domains for expending energy, including work, travel, recreation and domestic settings. Recent international measures have included estimates of total physical activity and have asked about the different domains in which adults can report activity. The most common measures are the International Physical Activity Questionnaire (IPAQ) and the Global Physical Activity Questionnaire (GPAQ) [14,15]. The short-form of IPAQ considers all domains collectively in generic questions, and has been used to compare prevalence rates within and between countries worldwide [16,17]. Given the length of the long form of IPAQ, its use for international comparisons has been less frequent. The intermediate-length Global Physical Activity Questionnaire (GPAQ) [15] was developed by the World Health Organization (WHO) to measure physical activity across the work, transport and recreation domains separately, and is used in many countries as part of the WHO STEPwise approach to Non-Communicable Disease surveillance (STEPS) [18]. Both IPAQ and GPAQ are reliable and valid measures [14,15,19]. Moreover, both IPAQ-long and GPAQ are suitable for use in developing countries where physical activity is more likely to be accrued across a range of domains [7,8].

The Asia-Pacific region comprises diverse, predominantly low-middle income countries (LMIC) [20] ranging from large and densely populated Asian countries to tiny Pacific island nations. Given the major increases in NCDs in the region [21,22], risk factor surveillance, including physical activity, is essential.

This paper has two purposes: i) to identify and compare published national physical activity prevalence estimates in adults aged 18-64 years in the Asia-Pacific countries in the WHO Western Pacific (WPRO) and South East Asian (SEARO) regions; ii) to examine variations in the prevalence and trends in physical activity within and between these countries, using different surveillance instruments.

## Methods

Data on physical activity prevalence across the Asia-Pacific region was obtained from several sources. A MEDLINE search used a combination of search terms including 'physical activity' or 'inactivity' or 'exercise', and 'prevalence', Asia, Pacific, Southeast Asia, Central Asia and individual country names. The full search strategy can be found in Additional file 1. Additional searching was carried out through the Asia-Pacific Physical Activity Network (AP-PAN) [23]; resources identified through AP-PAN and WHO links in the region included country-level health and NCD reports. Reports and websites of governmental and non-governmental organisations were also reviewed. This comprises a large amount of grey literature, not cited in indexed academic databases; however, this method generated many of the estimates used.

Relevant data were also extracted from sources including the World Health Organization's Global Health Infobase (STEPS) [18], the World Health Survey [16] the International Prevalence Study [17]. In some cases, authors were contacted for further information and raw data. Additional studies known to authors or AP-PAN members were obtained. Reference lists of retrieved studies were also reviewed.

Published literature and electronic resources accessed were limited to those in the English language. In order to profile recent epidemiology, only studies conducted between 2000 and 2010 were included. Where possible studies with sample sizes over 1000 were sought; however in some cases, nations with small populations that had conducted studies with fewer subjects were included. Where serial surveys using the same methodology were available, data prior to 2000 was included to establish physical activity trends. Where more than one survey was available for a country, all relevant data were included. This review was limited to nationally representative population-level studies or those reporting large-scale, sub-national population-level data from specific region(s). Studies involving children or adolescents or adults aged 65 years and above were excluded.

Physical activity estimates are presented as described in the original source documentation. For surveys where data were reported separately for males and females, the mean of these values was used as a population proxy estimate, as both genders tended to be proportionally represented or were weighted proportionally in samples. Some studies reported the percent achieving a threshold level 'sufficiently active' for health, defined as achieving 'at least 150 min of moderate-intensity aerobic physical activity throughout the week' [10]. Other studies reported mean or median MET-minutes per day of physical activity. MET-minutes are metabolic equivalents that describe the intensity of physical activity (amount of energy expended) relative to sitting quietly [24]. Where applicable, data obtained from other measures unique to individual surveys were also included.

## Results

A total of 56 surveys from 29 countries in the Asia-Pacific region were included in this review. Sample sizes ranged from 586 in the small Pacific island nation of Tokelau to 42,500 in India. Nationally representative samples were used in 37 studies (66%). Table 1 shows the physical activity prevalence data by country and outlines year of survey, sample size, representativeness and the domains assessed. A list of 12 countries where national or representative data was unavailable or unknown for the period 2000-2010 can be found in Additional file 2.

**Table 1:** Prevalence of physical activity in the Asia-Pacific region

Country	Survey	Year(s)	Sample size	Scope	Population Weighting	Domain	% sufficiently active	Median- or mean METmins/day	Other measures	Trends
2. Australia	Active Australia Survey [25]	1997	1997: 6803	National	Yes	L	1997: 62	N/A	N/A	^(1) ^↓over 3 yrs
									
		1999	1999: 3841				1999: 57			
									
		2000	2000: 3590				2000: 58			
						
	National Health Survey [26-28]	1995	1995: 39110		Yes	L	1995-6: 39			^(2) ^↔ over 10 yrs
									
		2000	2000: 17918				2000: 40			
									
		2004-5	2004/5: 19501				2004-5: 39			
						
	IPAQ Short Form [17]	2003	2691		Yes	all	83			^(3) ^N/A

3. Bangladesh	WHO NCD STEPS Survey (GPAQ) [29]	2002	11409	Sub-national	Yes	W, T	N/A	161 mean	N/A	N/A
					
	World Health Survey (IPAQ) [16]	2003	5166	National	Yes	all		131 median		

4. Cambodia	Diabetes and associated disorders in Cambodia [30]	2004	^(1) ^1195 (Kampong Cham region)	Sub-national	unknown	W, T, L	^(1) ^51	N/A	N/A	N/A
										
			^(2) ^1051 (Siem Reap region)				^(2) ^68			

5. China	InterASIA [31]	2000-1	15540	National	Unknown	W, L	66	N/A	^(1) ^N/A	N/A
		
	World Health Survey (IPAQ) [16]	2002	3596	Sub-national	Yes	all	90	697 median	N/A	
		
	Cardiovascular Health of residents: cities in China [32]	2001	2165	Sub-national	Unknown	L	N/A	6 mean	N/A	
		
	Chinese Third National Health Services Survey [33]	2003	19057	National	No	L	N/A	N/A	Regularly exercising: 13.6%	
		
	IPAQ Short Form [17]	2002	1593 (Shanghai)	Sub-national	Yes	all	93	N/A	N/A	
		
	IPAQ Short Form [17]	2002-3	4886 (SAR Hong Kong)	Sub-national	Yes	all	85	N/A	N/A	

6. Fiji	WHO NCD STEPS Survey (*adapted from *GPAQ) [34]	2002	6,783	National	Yes	W, T, L	N/A	N/A	Mod-high active W: 49.5%	N/A
										
									T: 85.1%	
										
									L: 24.0%	

7. India	WHO modified STEPS Survey (GPAQ) [35]	2003-5	42500	Sub-national	Yes	W, T, L	84	^(1) ^356 median	N/A	N/A
					
	World Health Survey (IPAQ) [16]	2003	7945		Yes	all	88	1461 median		
					
	IPAQ Short Form [17]	2003	1005		Yes	all	77	N/A		

8. Indonesia	Indonesia Health Survey 2001 [36]	2001	13131	National	Yes	W, T, L	36	N/A	N/A	N/A
			
	WHO STEPS survey (GPAQ) [37,38]	2003	2003: 1855	Sub-national	Yes	W, T, L	2003: 67 2006: 78	2003: 134 mean		↑over 3 yrs
									
		2006	2006: 1927					2006: W: 60		
										
								T: 26, L: 17 median		

9. Japan	National Sport Life Survey [39-42]	2000	2000: 2238	National	unknown	L	N/A	N/A	"Active Sport Participant" (> 2× wk, > 30 min, > moderate)	↓over 2 yrs then ↑over 2
									
		2002	2002: 2267							
									
		2004	2004: 2288							
									
		2006	2006: 1867							
										
									2000: 18%	
										
									2002: 13%	
										
									2004: 16%	
										
									2006: 16%	
			
	National Health and Nutrition Survey, Japan [18]	2000	2000: 6815	National	Unknown	unknown	N/A		"Inactive" (< 30 min, < 2× wk in last yr)	↔ over 3 yrs
									
		2001	2001: unknown							
									
		2002	2002: 9723							
									
		2003	2003: 9214						2000: 71%	
										
									2001: 72%	
										
									2002: 70%	
										
									2003: 77%	
			
	National Health and Nutrition Survey, Japan [18]	2004	2004: 4573	National	Unknown	unknown	21		N/A	N/A
			
	Fifth National Survey of CVD [18]	2000	8369	National	Unknown	unknown	N/A		< 4000 steps/day: 22%	N/A
			
	IPAQ Short Form [17]	2003	4959	Sub-national	Yes	all	57		N/A	N/A

Kiribati	WHO STEPS survey (GPAQ) [43]	2004-6	1,288	National	Yes	W, T, L	50	69 mean	N/A	N/A
										
								W: 39		
										
								T: 25		
										
								L: 5		

10. Republic of Korea	National Health & Nutrition Survey [44,45]	2001 (II)	2001: 7909	National	Yes	Unknown	2001: 28 2005: 22	N/A	N/A	↓over 4 yrs
									
		2005 (III)	2005: 7695							

11. Laos People's Democratic Republic	World Health Survey (IPAQ) [16]	2003	4640	National	Yes	all	88	143 median	N/A	N/A

12. Malaysia	WHO STEPS Survey (GPAQ) [46]	2005	3040	National	Yes	W, T, L	40	189 median	N/A	N/A
			
	World Health Survey (IPAQ) [16]	2003	5563	National	Yes	all	54	446 median		

13. Maldives	WHO STEPS Survey (GPAQ) [47]	2004	2000	Sub-national	Yes	W	7	N/A	N/A	N/A

14. Marshall Islands	WHO NCD STEPS Survey (GPAQ) [48]	2002	3045	National	Yes	W, T, L	34	W: 18 mean	N/A	N/A
										
								T: 45 mean		
										
								L: 12 mean		

15. Micronesia (Federated States of)	WHO NCD STEPS Survey (GPAQ) [49]	2005	1638	Sub-national	Yes	W, T, L	36	77 mean	N/A	N/A

16. Mongolia	WHO NCD STEPS Survey (GPAQ) [50]	2005	3411	National	Yes	W, T, L	89	189 median	N/A	N/A

17. Myanmar	WHO STEPS Survey (GPAQ) [51]	2003	2163	Sub-national (rural)	Yes	W, T, L	65	918 median	N/A	N/A
					
	WHO STEPS Survey (GPAQ) [52]		2285	Sub-national (urban)	Yes	W, T, L	51	288 median		
					
	World Health Survey (IPAQ) [16]		5517	National	Yes	all	91	694 median		

18. Nauru	WHO NCD STEPS Survey (GPAQ) [53]	2004	2272	National	Yes	W, T, L	N/A	203 median	17% physically inactive = no PA	N/A

19. Nepal	WHO STEPS Survey (GPAQ) [54]	2003	2030	Sub-national	Yes	W, T, L	18	N/A	% Inactive:	N/A
										
									W: 51	
										
									T: 19	
										
									L: 86	
		
	WHO STEPS Survey (GPAQ) [55]	2004-5	7792	Sub-national	Yes	W, T, L	N/A	450 median		
		
	World Health Survey (IPAQ) [16]	2003	7945	National	Yes	all	92	1351 median		

20. New Zealand	Sport and Physical Activity Survey [56]	1997/98	12500	National	Unknown	L	1997/98: 67	N/A	N/A	↑over 3 yrs
										
		1998/99					1998/99: 68			
										
		2000/01					2000/01: 70			
						
	New Zealand Health Survey [57,58]	2002-3	2002-3: 12929		Yes	L	2002-3: 52			↔ over 4 yrs
									
		2006-7	2006-7: 12488				2006-7: 51			
						
	IPAQ Short Form [17]	2003	1495		Yes	all	88			N/A

21. Pakistan	World Health Survey (IPAQ) [16]	2003	5610	National	Yes	All	80	623 median	N/A	N/A

22. Philippines	World Health Survey (IPAQ) [16]	2003	9535	National	Yes	All	93	1158 median	N/A	N/A

23. Samoa	WHO NCD STEPS Survey (GPAQ) [59]	2002	2817	National	Yes	W, T, L	50	W: 9 mean	N/A	N/A
										
								T: 3 mean		
										
								L: 2 mean		

24. Singapore	Singapore National Health Survey [60,61]	1992	1992: 3568	National	Unknown	L	1992: 14	N/A	N/A	↑over 12 yrs
									
		1998	1998: 4723				1998: 17			
									
		2004	2004: 4084				2004: 25			

25. Sri Lanka	WHO NCD STEPS Survey (GPAQ) [62]	2003	3,000	Sub-national	Yes	W, L	85	N/A	Mean mins spent in PA/day: 257	N/A
		
	World Health Survey (IPAQ) [16]	2002-3	5,464	National	Yes	All	90	1,089 median		

26. Taiwan	National Health Survey of Taiwan [63-66]	2001	15,559	National	unknown	L	^(1) ^21	N/A	^(1) ^N/A	^(1) ^N/A
					
	National Council on Physical Fitness and Sports [63,64]	2000	2000: 1021			L	^(2) ^N/A		^(2) ^> 3× wk, > 30 mins	^(2) ^↑over 3 yrs
									
		2001	2001: 8573						2001: 10%	
									
		2004	2004: 4073						2004: 14%	
					
	IPAQ Short Form [17]	2004	4846		Yes	all	^(3) ^58		^(3) ^N/A	^(3) ^N/A

27. Thailand	Adapted IPAQ (long form) (2003) IPAQ short version (2004-6) [65]	2003	2003: 11462	National	unknown	all	2003: 59	N/A	N/A	↑over 3 yrs
									
		2004	2004: 8383				2004: 79			
									
		2006	2006: 15158				2005: 83			
										
							2006: 79			

28. Tokelau	WHO NCD STEPS Survey (GPAQ) [66]	2005	586	National	Yes	W, T, L	58	111 mean	N/A	N/A

29. Vietnam	WHO NCD STEPS Survey (GPAQ) [7]	2005	1906	Sub-national	Yes	W, T, L	56	N/A	N/A	N/A
			
	World Health Survey (IPAQ) [16]	2002-3	3009	National	Yes	All	92	98 median		

*WHO NCD STEPS *WHO STEPwise approach to Non-Communicable Disease surveillance, *InterASIA *International Collaborative Study of Cardiovascular Disease in Asia, *GPAQ *Global Physical Activity Questionnaire, *IPAQ *International Physical Activity Questionnaire, *W *Physical activity in the 'work' domain, *T *Physical activity in the 'travel' domain, *L *Physical activity in the 'leisure' domain, ↑ Increase, ↔ No substantial change, ↓ Decrease, *N/A *Not applicable, *II *Second survey version, *III *Third survey version

Nineteen surveys reported data on median MET-minutes/day, with estimates ranging from 98 in Vietnam to 1461 in India. Six surveys contained information on mean MET-minutes, ranging from 6 in China to 161 in Bangladesh. Additionally, in Indonesia, median MET-minutes/day were reported according to domain of physical activity (work: 60 MET-minutes; transport: 26 MET-minutes; leisure: 17 MET-minutes) and three countries reported mean MET-minutes by domain. In the Marshall Islands, the travel domain contributed 45 MET-minutes compared with work (18 MET-mins) and leisure (12 MET-mins) domains. In Kiribati and Samoa, the work domain contributed most (39 and 9 MET-minutes respectively) to the total, followed by travel (25 and 3 MET-minutes respectively) and leisure (5 and 2 MET-minutes respectively) domains.

Surveys other than the IPAQ or GPAQ were reported from nine countries. Six of these countries used multiple additional survey instruments, totalling 18 studies using other methodologies. Nine of these surveys (16% of the total sample) were national health surveys, three (5%) were national sport surveys and the remaining six (11%) used unique measurement tools. Ten surveys measured the percent of sufficiently active adults, with estimates varying from 14% in Singapore to 70% in New Zealand.

Comparisons can be made among surveys that used the same measures. Nineteen of the surveys (34%) used the IPAQ, as part of the World Health Survey. Estimates of the proportion 'sufficiently active' ranged from 54% in Malaysia to 93% in the Philippines (median 90%, inter-quartile range 80-92%). Median MET-minutes of activity varied from 98 in Vietnam to 1461 in India (median 694 MET-minutes, inter-quartile range 143-1156).

Eighteen surveys (37%) used the GPAQ, as part of the WHO NCD STEPS Surveys. Fifteen of these provided estimates of the percent 'sufficiently active', ranging from 7% in Maldives to 89% in Mongolia (median 53.5% inter-quartile range 44.5-80.5). Ten surveys provided median MET-minutes, ranging from 134 in Indonesia to 918 in Bangladesh and Myanmar and five provided mean MET-minutes ranging from 69 in Kiribati to 111 mean MET-minutes in Tokelau.

Eighteen surveys provided data on both percent 'sufficiently active', and median and/or mean MET-minutes of physical activity. For the majority of these surveys, both measures were similar. For example, in the Philippines where 93% were 'sufficiently active', the median activity was 1158 MET-minutes/day. However, in Vietnam, the percent 'sufficiently active' was high at 92% but the median activity was discordant at only 98 MET-minutes.

Of the 56 surveys reported here, 11 (22%) were conducted more than once providing trend information. Of these, five countries (Indonesia, New Zealand, Singapore, Taiwan, and Thailand) demonstrated increases in physical activity levels over time. Three surveys conducted in Australia, the Republic of Korea and Japan demonstrated decreases in physical activity levels over time. The remaining three from Australia, Japan and New Zealand did not show significant changes in either direction over time (data not shown).

**Figure 1:**
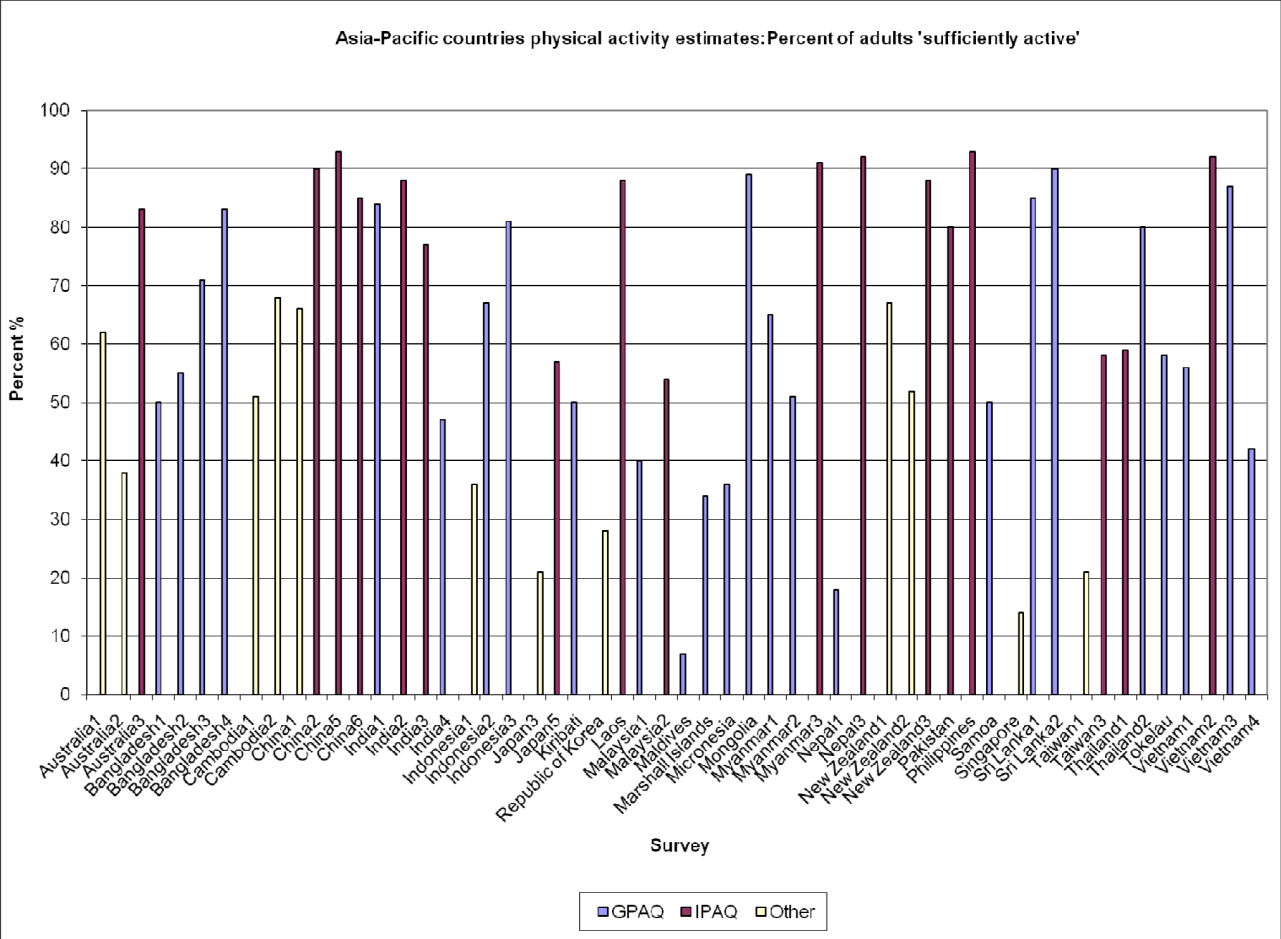
**Asia-Pacific countries physical activity estimated: Percent of adults 'sufficiently active'**.

Figure 1 displays estimates from the 45 surveys (80%) providing data on "sufficiently active" adults in the population, ranging from 7% in the Maldives to 93% in the Philippines (median estimate 62%, inter-quartile range 40-85). In 14 countries more than one survey has been used. Six such nations reported prevalence data from both IPAQ and GPAQ and in all these cases, higher proportions of 'sufficient activity' were reported using the IPAQ measure compared with the GPAQ. The greatest variation was in Nepal; with estimates of 18% sufficiently active obtained using GPAQ, compared with an estimate of 92% from IPAQ.

Surveys that used other instruments to examine leisure-time physical activity reported substantially lower prevalence estimates compared with countries using the multi-domain measures of IPAQ and/or GPAQ. Similar differences in point estimates derived from IPAQ and GPAQ are evident for median MET-minutes, with three of the four countries with data from both surveys reporting higher values using IPAQ than GPAQ. This discrepancy was most pronounced in India, with estimates varying from 1461 median MET-minutes from IPAQ to 356 median MET-minutes from GPAQ. Only Myanmar reports a higher median MET-minutes value using GPAQ than IPAQ (918 compared with 694).

## Discussion

This paper is a review of published population-level physical activity estimates among adults in the Asia-Pacific region. Standardised and comparative physical activity prevalence information from other regions currently exists, such as the IPAQ survey conducted in 51 countries through the 2002 World Heath Survey [16] and the 20-country International Prevalence Study [17]. Whilst these studies give estimates of physical activity prevalence in countries using the same measure and similar protocols, unexpected results in some countries are apparent. Our paper is the first to report prevalence data from many countries that have collected information using several instruments including IPAQ, GPAQ and other leisure-time physical activity surveys. It highlights the large variation between estimates obtained using different measurement tools, and thus demonstrates the need for standardisation of samples and of measures.

This review has demonstrated that physical activity estimates vary widely, even within a single country using different surveys over similar time periods. For example, with the exception of India and Sri Lanka, 11 of the 13 countries with more than one survey reporting the percentage sufficiently active give substantially different estimates from different surveys. Similarly, all four countries reporting values for median MET-minutes from different surveys showed substantial variation in reported physical activity.

Notably, household and garden activities are included in GPAQ but for the remainder of surveys using alternative instruments, physical activity in this domain was unknown. Consequently, there may be an underestimation of overall activity level. This is particularly relevant to developing countries in the Asia-Pacific region, where this domain may contribute a higher proportion to total than in more industrialised countries. Careful consideration should be given to the need to measure physical activity in different domains, and the consequences of these methodological decisions on prevalence estimates.

In developing countries outside the Asia-Pacific region, a similar situation regarding physical activity surveillance measures is evident. In two Brazilian studies which both used the multi-domain IPAQ long-form questionnaire, the prevalence of inactive adults was reported as 41.1% in a Brazilian city survey [67] and 26.1% inactive in a national sample [14]. A third Brazilian study [68] reports that only 3% of adults were sufficiently active in leisure-time alone, compared with the higher estimates derived from using the more generic IPAQ measure. Similarly, in Saudi Arabia, one estimate [69] gives high *inactivity *prevalence of 96% in a large-scale national survey examining leisure-time activity, yet another [8] reports only 41% inactive using IPAQ in over 1000 adults living in the capital city. These studies confirm the large prevalence differences between total physical activity measures and leisure time-only surveys.

Physical activity is an important indicator for the health and sport sectors, and also for other sectors such as transport and urban planning. This is the first time physical activity estimates have been collated among adult populations, especially in LMIC. The policy and practical implications of this work are profound, particularly in the setting of burgeoning obesity prevalence and the NCD epidemic confronting many Asia-Pacific nations. Accurate and consistent measurement of physical activity is essential to guide evidence-based public health practice and facilitate and target health promotion initiatives. Moreover, accurately documenting participation in various domains of physical activity may facilitate a multi-sectoral 'health in all policies' approach.

Whilst our criteria for selection of studies were carefully considered, the use of grey literature can be problematic. The true denominator of actual studies cannot be ascertained, so this is a collection of estimates. Thorough review and contact with national agencies led us to consider this a comprehensive distillation, but the epithet 'systematic review' cannot be attached to these methods. Additionally, reliability and validity of instruments reported in the grey literature is unknown, potentially limiting their accuracy. Nonetheless, since most of the international physical activity measures from LMIC are reported in the grey literature, this is a large collection which identifies important issues in physical activity surveillance and estimation, within and between countries. It also identifies problems that might be caused by incompatible surveillance systems. Whilst some of the variation seen in this review reflects real differences in physical activity, other factors such as the domains of activity included in the survey questionnaire and population sampling differences, may have influenced these results. Even using the same survey and protocol, countries similar in size, culture or stage of development may show unexplained differences in prevalence [16]. Assessing reliability and validity of existing measures in the specific population where measurement is intended is advised. More intensive research, using a mix of qualitative and quantitative methodologies, may also be needed to understand and explain observed differences, prior to further surveillance.

Included in this research agenda is population-level physical activity monitoring in the countries listed in Additional file 2 where data was unavailable or unknown for the period 2000-2010, as well as the need for sub-samples to be compared with objective physical activity measures, using accelerometry or pedometers at the population level [70-72]. These objective studies point to over-estimation of self-report physical activity levels, which may mean that current burden of disease calculations are under-estimating the population attributable fraction for inactivity. Nonetheless, even in the absence of objective surveillance, physical activity prevalence across the Asia-Pacific region should be compared using identical sampling methods across surveys and using established, reliable and valid measures.

## Conclusions

There is an urgent need for standardization of physical activity measures and survey methods used within and between countries, in order to accurately document risk factor prevalence and population trends. Between-country comparisons are also easier when methodologically comparable data collection techniques are adopted, and when identical protocols and analytic methods are used. It is encouraging that many developing countries collect physical activity survey data, either as part of NCD surveillance or from other sectors, and report on population participation rates, active transport, or total physical activity. This review highlights differences between estimates and demonstrates that use of different questionnaires, even within the same country, may yield discrepant results. The challenge in public health surveillance is to work towards comparability of measures and methods to assess physical activity in populations.

## Competing interests

The authors declare that they have no competing interests.

## Authors' contributions

RM and MA participated in data collection and synthesis. AB conceived of the study and participated in its design. All authors helped draft and approved the final manuscript.

## Pre-publication history

The pre-publication history for this paper can be accessed here:

http://www.biomedcentral.com/1471-2458/12/41/prepub

## Supplementary Material

Additional file 1 **Search Terms Used In Medline Search For Asia Pacific Physical Activity Data**. Click here for file

Additional file 2 **Countries where national or representative data was unavailable or unknown for the period 2000-2010**. Click here for file
