# Supplementary material for: A review of population-based prevalence studies of physical activity in adults in the Asia-Pacific region
Source: BMC Public Health. 2012 Jan 17;12:41. doi: 10.1186/1471-2458-12-41 (PMC3293715; doi:10.1186/1471-2458-12-41)
Supplement: Additional file 1 — Search Terms Used In Medline Search For Asia Pacific Physical Activity Data. [file 1471-2458-12-41-S1.DOCX]

**Appendix 1**

**Search Terms Used In Medline Search For Asia Pacific Physical Activity Data**

1. physical activity.mp.

2. Leisure Activities/ or Exercise/ or physical inactivity.mp.

3. 1 or 2

4. Prevalence/

5. Asia, Central/ or Asia/ or Asia, Western/ or Asia, Southeastern/

6. 4 and 3

7. American Samoa/ or Pacific Islands/

8. Australia/

9. Bangladesh/

10. China/

11. Fiji/

12. India/

13. Indonesia/

14. Japan/

15. Laos/

16. Malaysia/

17. maldives.mp. or Indian Ocean Islands/

18. Mongolia/

19. Myanmar/

20. nauru.mp. or Micronesia/

21. Nepal/

22. New Zealand/

23. Pakistan/

24. Philippines/

25. "Independent State of Samoa"/ or American Samoa/ or Samoa/

26. Singapore/

27. Sri Lanka/

28. Taiwan/

29. Thailand/

30. tokelau.mp. or Polynesia/

31. Vietnam/

32. 11 or 21 or 7 or 26 or 17 or 22 or 18 or 30 or 23 or 16 or 13 or 29 or 27 or 25 or 28 or 9 or 12 or 14 or 15 or 20 or 8 or 24 or 10 or 19 or 31 or 5

33. 6 and 32
