# Supplementary material for: A review of population-based prevalence studies of physical activity in adults in the Asia-Pacific region
Source: BMC Public Health. 2012 Jan 17;12:41. doi: 10.1186/1471-2458-12-41 (PMC3293715; doi:10.1186/1471-2458-12-41)
Supplement: Additional file 2 — Countries where national or representative data was unavailable or unknown for the period 2000-2010. [file 1471-2458-12-41-S2.DOCX]

**Additional file 2: Countries where national or representative data was unavailable or unknown for the period 2000-2010**

Bhutan
Brunei Darussalam
Cook Islands
Democratic People’s Republic of Korea
Myanmar
Niue
Palau
Papua New Guinea
Solomon Islands
Timor-Leste
Tonga
Tuvalu
